# Association between dysglycemia and mortality by diabetes status and risk factors of dysglycemia in critically ill patients: a retrospective study

**DOI:** 10.1007/s00592-021-01818-3

**Published:** 2021-11-11

**Authors:** Haoming Ma, Guo Yu, Ziwen Wang, Peiru Zhou, Weitao Lv

**Affiliations:** 1 School of Nursing, Jinan University, No. 601, West Huangpu Avenue, Tianhe District, Guangzhou City, Guangdong Province China; 2 Health Management Centre, The First Affiliated Hospital of Jinan University, No. 613, West Huangpu Avenue, Tianhe District, Guangzhou City, Guangdong Province China; 3 Division of Critical Care, The First Affiliated Hospital of Jinan University, No. 613, West Huangpu Avenue, Tianhe District, Guangzhou City, Guangdong Province China

**Keywords:** Dysglycemia, Diabetes mellitus, Mortality, ICU, Critically ill, Risk factors

## Abstract

**Aims:**

Dysglycemia, including the three domains hyperglycemia, hypoglycemia, and increased glycemic variability (GV), is associated with high mortality among critically ill patients. However, this association differs by diabetes status, and reports in this regard are limited. This study aimed to evaluate the associations between the three dysglycemia domains and mortality in critically ill patients by diabetes status and determined the contributing factors for dysglycemia.

**Methods:**

This retrospective study included 958 critically ill patients (admitted to the ICU) with or without DM. Dysglycemia was defined as abnormality of any of the three dimensions. We evaluated the effects of the three domains of glucose control on mortality using binary logistic regression and then adjusted for confounders. The associations between dysglycemia and other variables were investigated using cumulative logistic regression analysis.

**Result:**

GV independently and similarly affected mortality in both groups after adjustment for confounders (DM: odds ratio [OR], 1.05; 95% confidence interval [CI]: 1.03-1.08; *p* <0.001; non-DM: OR, 1.07; 95% CI, 1.03-1.11; *p* = 0.002). Hypoglycemia was strongly associated with ICU mortality among patients without DM (3.12; 1.76-5.53; *p* <0.001) and less so among those with DM (1.18; 0.49-2.83; *p* = 0.72). Hyperglycemia was non-significantly associated with mortality in both groups. However, the effects of dysglycemia seemed cumulative. The factors contributing to dysglycemia included disease severity, insulin treatment, glucocorticoid use, serum albumin level, total parenteral nutrition, duration of diabetes, elevated procalcitonin level, and need for mechanical ventilation and renal replacement therapy.

**Conclusion:**

The association between the three dimensions of dysglycemia and mortality varied by diabetes status. Dysglycemia in critical patients is associated with excess mortality; however, glucose management in patients should be specific to the patient’s need considering the diabetes status and broader dimensions. The identified factors for dysglycemia could be used for risk assessment in glucose management requirement in critically ill patients, which may improve clinical outcomes.

## Background

Most studies have reported on the high incidence of dysglycemia (including the 3 dimensions hyperglycemia, hypoglycemia, and increased glycemic variability [GV]) and its independent association with mortality among critically ill patients [1–6]. Recent studies have shown that diabetes status modulates the association between these glycemia domains and mortality or other important clinical outcomes during intensive care unit (ICU) hospitalization [7–12].

The associations between mortality and the 3 domains of dysglycemia have been reported to vary significantly between patients with or without diabetes mellitus (DM) [7, 13]. Recent observational studies suggest that those with pre-existing diabetes present a “blunted effect” to increased GV and hyperglycemia. This has been attributed to the likely higher tolerance to acute glucose fluctuation in patients with DM [13–15]. However, some recent studies have reported that the association between hypoglycemia and mortality is stronger in patients with DM [7], which is inconsistent with the findings of other studies. Moreover, most of these studies did not consider comorbidities, disease severity, inflammation level, insulin therapy and other in ICU treatments (including glucocorticoid use and nutrition therapy), which known as confounding factors when estimating the effects of glucose metrics [16].

Therefore, the associations between different glycemic metrics and mortality in ICU remain unclear, and studies directly and quantitatively comparing the effects of these glucose control domains in critically ill patients with comprehensive adjustments for confounders are scarce [17]. Because the complex association between ICU patients’ outcomes and GV depends on DM, determining the effects of different glycemic metrics in DM and non-DM patients is important.

In this context, we hypothesized that the effects of the 3 domains of dysglycemia on mortality would differ between patients with or without DM. Therefore, we conducted a retrospective study using data of ICU patients to, primarily, explore the effect of different glycemic metrics on mortality and further identify risk factors for dysglycemia in our patient sample.

## Methods

### Description of study design and patient enrollment criteria

This retrospective observational study used electronic clinical data from patients admitted to the ICU of the First Affiliated Hospital, Jinan University between January 1, 2019, and December 31, 2020. The institute’s ICU is a mixed medical and surgical ICU. Blood glucose level was measured using a glucometer, and measurements were fed into an interactive database.

Patients admitted during the study period who were aged ≥ 18 years and were treated in the ICU for ≥ 24 h were eligible. Exclusion criteria included (1) incomplete mortality data; (2) ICU hospitalization < 24 h; (3)  < 8 blood glucose measurements on the first day after admission; (4) treatment discontinuation by family; (5) hospital stay > 120 days; (6) hospitalization for treatment of diabetic ketoacidosis or diabetic hyperglycemic hyperosmolar state.

### Clinical data gathered

We obtained clinically relevant information from the ICU database and the hospital electronic medical record system. Measures extracted for the analyses included baseline demographics (age, sex, patient diagnosis, and comorbidities, past medical history, severity of illness score [APACHE II score]); ICU treatments (exogenous insulin, nutrition type and steroid use); clinical information (length of ICU stay [ICU-LOS], total length of hospital stay [LOS], blood glucose level, hemodialysis duration in hours, duration of mechanical ventilation, other biochemical indicators, and patient outcomes).

### Blood glucose and nutrition management protocol

According to the guidelines of the American Diabetes Association [18], the target blood glucose range for critically ill patients was 7.8–10.0 mmol/L (140–180 mg/dL) in our study. A standard blood glucose control goal and a uniform insulin infusion standard to maintain the blood glucose level within the target range were adopted. The nutrition therapy emphasized on early enteral nutrition, starting feeding within 48 h, and attempting to achieve sufficient nutritional support, in accordance with the recommendations of the ICU nutritional therapist, within 48–72 h [19].

Capillary blood glucose levels were used in this study. The glucose level was measured uniformly by nurses using a glucose meter (FreeStyle Optium Blood Glucose and Ketone Monitoring System, Abbott Diabetes Care, Oxon, UK). Glucose level measurements were made at least every 4 h after admission to the ICU and for no less than 6 times per day.

### Variable definitions

Patients were assigned to the DM or non-DM group according to the relevant clinical data obtained from patients, family members, and the documentation in their electronic medical records. We also collected the duration of DM (years) for the DM group, which was defined as the time (years) since the first diagnosis of diabetes. Disease severity was assessed using the APACHE II scores [20]. Blood glucose levels during ICU hospitalization were retrieved electronically from the relevant data storage unit, and we used Microsoft Excel software (Microsoft® Excel® 2016 MSO 16.0.14131.20278) to calculate the coefficient of variation (CV), mean blood glucose (MBG), and standard deviation (SD) for each patient.

We defined the glycemic metrics basis prior studies [21–23]. GV was expressed as the CV of blood glucose in this study [24]. CV is defined as the SD of blood glucose divided by the corresponding MBG (SD\MBG × 100). We defined increased GV as CV ≥ 20%, hypoglycemia as at least 1 BG concentration < 3.9 mmol/L (70 mg/dL), and hyperglycemia as mean glucose concentration > 7.8 mmol/L for non-DM patients and > 10 mmol/L for DM patients. Furthermore, dysglycemia was defined as abnormality of any of the 3 dimensions of glucose management (high BG, low BG, and BG excursion). The factors associated with dysglycemia in each patient were defined as ordinal categorical variables, wherein “0” referred to no dysglycemia and “3” to the presence of all 3 domains of dysglycemia.

### Outcome measures and adjustment for confounders

Hospital mortality, defined as death before hospital discharge, was the main outcome measure in this study. The secondary outcome measure was dysglycemia during ICU stay, including the 3 dimensions of poor glucose management (hyperglycemia, hypoglycemia, increased GV).

Variables considered for adjustment when determining the effect of dysglycemia on adverse outcomes in ICU patients included basic patient information (sex, age, disease severity [basis APACHE II score], MBG, GV [basis CV], hyperglycemia, hypoglycemia); laboratory data on admission to ICU (white blood cell count, hemoglobin, serum albumin, serum creatinine, and procalcitonin levels); comorbidities (hypertension, coronary heart disease, cerebral infarction, and chronic kidney disease); in ICU treatment (duration of ventilation in hours, duration of hemodialysis in hours, insulin treatment, corticoid use, and nutrition therapy).

### Data analysis strategy

First, we assessed the data distribution. For continuous variables, we used means ± standard deviations for normally distributed ones and medians and interquartile ranges (25% quartile to 75% quartile) for non-normally distributed ones. Categorical variables were presented as percentages.

For between-group comparisons of continuous variables, the t-test was applied to compare the normally distributed variables, whereas the Mann-Whitney rank-sum test was used for non-normally distributed ones. The chi-square test was used to compare categorical variables between groups, and the Bonferroni test was used for pairwise comparisons between groups of 3 or more.

Our patient samples were stratified into 2 groups: critically ill patients with diabetes (DM group) and without diabetes (non-DM group). We performed logistic regression analysis in 2 groups to adjust for age, sex, APACHE II score, comorbidities and laboratory data when estimating the different effects of 3 glycemic metrics (hyperglycemia, hypoglycemia, and glycemic variability) on mortality. For further control of confounders, we adjusted ICU treatment (insulin infusion therapy, glucocorticoid use, duration of ventilation and duration of hemodialysis) and the other two domains of glucose metrics.

The associations between dysglycemia and other variables were investigated using a cumulative logits model, which is suitable for analysis when the dependent variable was an ordinal categorical variable. All significant risk factors (*p* < 0.2) identified in the univariate analysis were considered as potential predictors and then entered into an initial multivariate model. We use forward stepwise selection to enter the independent variables into the model. A 2-sided *p* < 0.05 was considered statistically significant. Estimation of effects from the models devised are presented as odds ratios (ORs) and the corresponding 95% confidence intervals (CIs). We used SPSS version 17.0 software (SPSS Inc., Chicago, IL) for statical analysis.

## Results

### Clinical characteristics

**Fig. 1:**
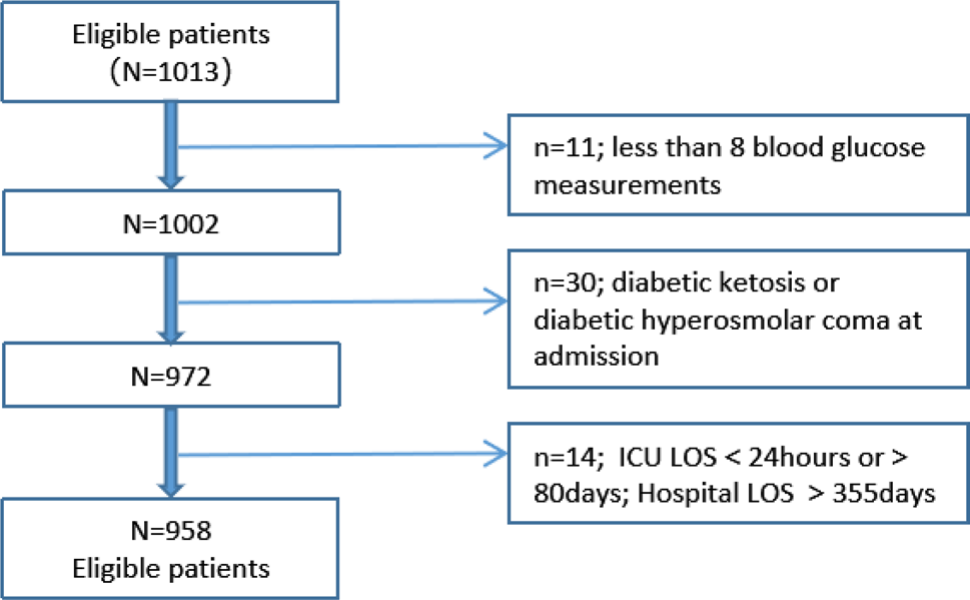
Patient flow diagram

This study included 958 critically ill patients. Figure 1 presents a flowchart for patient screening. Table 1 summarizes the key clinical characteristics of the patients, categorized by ICU mortality. Briefly, the mean patient age was 62.48 ± 17.86 years, 613 (64.0%) patients were male, and 238 (24.8%) patients had DM. The diagnosis in ICU admissions were 245 (25.6%) for surgical reasons and 713 (74,4%) for medical reasons, with mean APACHE II score was 25.61 ± 9.15 at admission. At the end of the study period, mortality rate of our unit was 34,2%. The most common underlying comorbidities were hypertension 428 (44.7%), DM 238 (24.8%), cerebral infarction 155 (16.2%), coronary heart disease146 (15.2%) and chronic kidney disease 134 (14.0%). In addition, among patients who died during hospitalization, their glycemic metrics tended to be elevated, such as CV (29.36 ± 10.07 vs 22.14 ± 7.58; *p* < 0.001), MBG (10.78 ± 2.92 vs 9.99 ± 2.56; *p* < 0.001), and the incidence of hyperglycemia (81.7% vs 76.0%; *p* = 0.04) or hypoglycemia (35.7% vs 9.7%; *p* < 0.001) was higher (Table 1).

**Table 1:** Patient characteristics

	All patients *n* = 958	Survivors *n* = 630	Dead *n* = 328	P value for survivors vs dead in ICU
Basic information and glycemia data				
Age (years)	62.48 ± 17.86	63.07 ± 17.92	61.34 ± 17.73	0.154
Male n/N (%)	613/958 (64.0)	407/630 (64.6)	206/328 (62.8)	0.79
APACHE II score	25.61 ± 9.15	22.85 ± 8.10	30.93 ± 8.69	< 0.001
MBG(mmol/L)	10.26 ± 2.71	9.99 ± 2.56	10.78 ± 2.92	< 0.001
SD	2.61 ± 1.43	2.28 ± 1.15	3.25 ± 1.69	< 0.001
CV	24.58 ± 9.15	22.14 ± 7.58	29.36 ± 10.07	< 0.001
Hyperglycemia	747/958 (78.0)	479/630 (76.0)	268/328 (81.7)	0.044
Hypoglycemia	178/958 (18.6)	61/630 (9.7)	117/328 (35.7)	< 0.001
Comorbidities				
DM (%)	238/958 (24.8)	145/630 (23.0)	93/328 (28.4)	0.07
Hypertension (%)	428/958 (44.7)	263/630 (41.7)	165/328 (50.3)	0.011
CI(%)	155/958 (16.2)	92/630 (14.6)	63/328 (19.2)	0.066
CKD (%)	134/958 (14.0)	74/630 (11.7)	60/328 (18.3)	0.006
CHD (%)	146/958(15.2)	80/630(12.7)	66/328(20.1)	0.002
Clinical data				
HOSP-LOS (days)	16 (9–28)	18 (11–30)	11 (5–23)	< 0.001
ICU-LOS (days)	5 (2–11)	4 (2–11)	6 (3–12)	0.103
Duration of ventilation (hours)	24 (0–105)	8 (0–63)	67 (20–171)	< 0.001
Duration of hemodialysis (hours)	0 (0–8)	0 (0–4)	0 (0–16)	< 0.001
In ICU treatment				
Insulin infusion therapy (%)	372/958 (38.8)	210/630 (33.3)	162/328 (49.4)	< 0.001
Use of corticoids (%)	381/958 (39.8)	236/630 (37.5)	145/328 (44.2)	0.043
TEN (%)	68/958 (7.1)	52/630 (8.3)	16/328 (4.9)	0.054
TPN (%)	391/958 (40.8)	255/630 (40.5)	136/328 (41.5)	0.768
EN + PN (%)	499/958 (52.1)	323/630 (51.3)	176/328 (53.7)	0.483
Laboratory data				
WBC (× 10^9^/L)	12.7 (8.7–17.9)	12.6 (8.8–17.3)	13.0 (8.1–19.1)	0.66
HGB (g/L)	103.3 ± 31.7	105.6 ± 31.4	98.8 ± 32.0	0.002
ALB (g/L)	29.5 ± 6.7	30.2 ± 6.6	28.2 ± 6.6	< 0.001
CREA (umol/L)	105.0 (72.0–196.0)	93.5 (68.8–168.0)	127.0 (87.0–246.5)	< 0.001
PCT (ng/ml)	1.6 (0.5–14.4)	1.2 (0.4–9.7)	3.5 (0.7–24.0)	< 0.001

Data are displayed as *n* (%), median (25th–75th percentiles), or mean ± SD, unless otherwise indicated. MBG, mean blood glucose; SD, standard deviation; CV, coefficient of variation; CI, cerebral infarction; CKD, chronic kidney disease; CHD, coronary heart disease; TEN, total enteral nutrition; TPN, total parenteral nutrition; EN + PN, combined enteral nutrition and parenteral nutrition; WBC, white blood cell count; HGB, hemoglobin; ALB, albumin; CREA, creatinine; PCT, procalcitonin

### Association between dysglycemia and mortality

We used logistic regression to investigate the effects of the 3 domains of glucose control on mortality in DM and non-DM patients and further tightly control the confounding factors, as summarized in Table 2. Information on sex, age, APACHE II score, glycemic metrics, in ICU treatment, comorbidities, nutrition therapy and laboratory data was available for 958 patients.

**Table 2:** Associations of glycemic metrics with mortality in ICU patients with and without DM

		OR (95% CI) for mortality			
		Crude model	Model 1	Model 2	Model 3
DM	CV	1.09 * (1.04–1.13)	1.07 * (1.03–1.11)	1.07 * (1.02–1.11)	1.06 * (1.02–1.11)
	Mean BG	1.08 (0.98–1.20)	1.07 (0.96–1.19)	1.06 (0.94–1.19)	1.12 (0.95–1.31)
	Hypoglycemia < 3.9 mmol/L	3.01 * (1.49–6.12)	2.22 * (1.04–4.77)	2.10 (0.97–4.58)	1.18 (0.49–2.83)
	Hyperglycemia > 10 mmol/L	1.31 (0.67–2.55)	1.08 (0.52–2.24)	0.93 (0.43–2.00)	0.42 (0.14–1.22)
Non-DM	CV	1.11*(1.08–1.13)	1.08*(1.06–1.11)	1.08*(1.05–1.10)	1.05*(1.02–1.08)
	MBG	1.13 * (1.06–1.20)	1.07 (0.99–1.14)	1.03 (0.95–1.11)	1.06 (0.96–1.15)
	Hypoglycemia < 3.9 mmol/L	6.25 * (4.19–9.33)	5.09 * (3.25–8.00)	4.65 * (2.93–7.39)	3.12 * (1.76–5.53)
	Hyperglycemia > 7.8 mmol/L	1.42 (0.97–2.10)	0.97 (0.62–1.50)	0.89 (0.56–1.43)	0.87 (0.48–1.58)

Model 1: adjusted for age, sex and APACHE II score

Model 2: adjusted for variables in model 1 plus comorbidities, laboratory data, insulin infusion therapy (yes/no), glucocorticoid use (yes/no), duration of ventilation (hours) and duration of hemodialysis (hours)

Model 3: model 2 adjustments plus other glucose metrics (for example, to estimate the effect of CV in DM group, we adjusted for MBG, hypoglycemia, and hyperglycemia > 10 mmol/L)

*p ≤ 0.05

In the DM group, higher levels of GV (OR, 1.09; 95% CI, 1.04–1.13) and a higher rate of hypoglycemia (OR, 3.01; 95% CI, 1.49–6.12) were significantly associated with a higher risk of mortality in the initial model (crude model). The effect of GV on mortality was relatively stable after adjustments for all the confounders (model 1: 1.07, 1.03–1.11; model 2: 1.07, 1.02–1.11; model 3: 1.06, 1.02–1.11). However, the association between hypoglycemia and mortality weakened and became nonsignificant after adjusting for ICU treatment and other glucose metrics (model 2: 2.10, 0.97–4.58 [*p* = 0.06]; model 3: 1.35, 0.55–3.33 [*p* = 0.51]). In addition, although MBG and hyperglycemia were regarded as potential risk factors for mortality, the effects of MBG and hyperglycemia on mortality were nonsignificant in all models.

In the non-DM group, the effects of GV (OR, 1.11; 95% CI, 1.08–1.13; *p* < 0.001), MBG (OR, 1.13; 95% CI, 1.06–1.20; *p* < 0.05) and hypoglycemia (OR, 6.25; 95% CI, 4.19–9.33; *p* < 0.001) on mortality were significant in the crude model. Adjusting for disease severity and other risk factors slightly decreased the ORs for hypoglycemia (model 1: OR, 5.09; 95% CI, 3.25–8.00; model 2: OR, 4.65; 95% CI, 2.93–7.39) but not for GV (model 1: OR, 1.08; 95% CI, 1.06–1.11; model 2: OR, 1.08; 95% CI, 1.05–1.10) even after adjustment for other glycemic metrics (OR, 1.05; 95% CI, 1.02–1.08). MBG showed a positive correlation with mortality in the initial model; however, it was nonsignificant after adjustment for basic information (sex, age, and APACHE II score). In addition, the effects of GV and hypoglycemia on mortality were attenuated yet significant after adjusting for other glycemic metrics, insulin infusion therapy, glucocorticoids use, duration of ventilation, and duration of hemodialysis. Hyperglycemia had no significant effect on mortality in all models, as in the DM group.

**Fig. 2:**
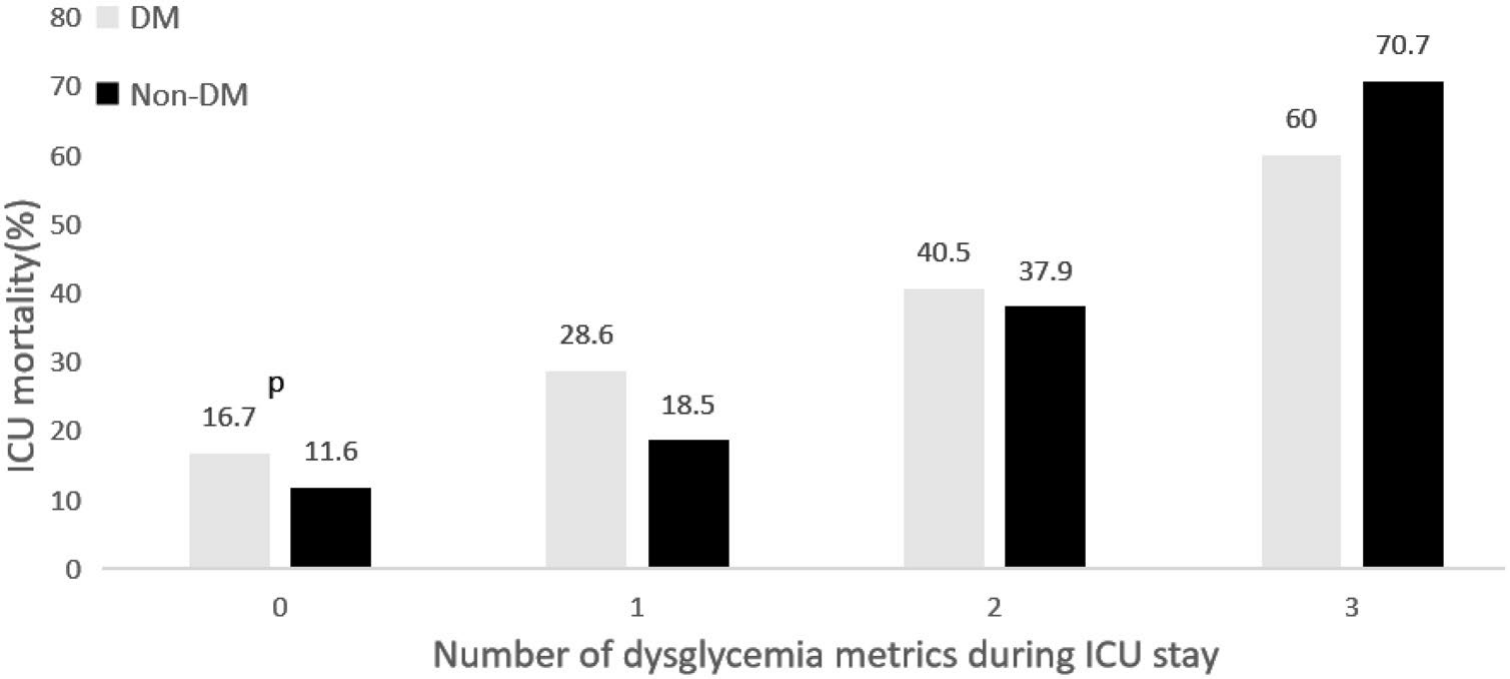
The cumulative effect of the number of dysglycemia metrics in ICU on mortality, categorized by the presence of diabetes

Of note, the effect of hypoglycemia in the non-DM group (OR, 3.12; 95% CI, 1.76–5.53) was stronger than in the DM group (OR, 1.35; 95% CI, 0.55–3.33) after adjustment for other confounders. However, the effect of GV on mortality was notably similar in both groups. Although hyperglycemia showed nonsignificant effects on mortality in both groups in the final logistic regression model, we found that the 3 domains had a cumulative effect on mortality (Fig. 2). The mortality rate was the lowest among patients without dysglycemia and diabetes and the highest among patients with abnormality across the 3 domains of dysglycemia and without diabetes. In addition, regardless of diabetes status, the mortality rate increased proportionally with the degree of dysglycemia.

Multivariate analysis (Table 2; Fig. 2) showed that in the non-DM group, compared to those without any domains of dysglycemia (no hyperglycemia [MBG ≤ 7.8 mmol/L for non-DM and ≤ 10 mmol/L for DM], no hypoglycemia episodes, and CV < 20%), the cohort with 3 domains of dysglycemia had an approximately sixfold higher mortality rate. Furthermore, these associations appeared to weaken for the DM cohort, with an approximately threefold mortality increase.

### Factors associated with dysglycemia

To identify the independent factors associated with dysglycemia, we devised a cumulative logistic regression model. As summarized in Table 3, in a multivariate analysis adjusted for sex and age, APACHE II score (adjusted odds ratio [AOR]), 1.06; 95% CI, 1.05–1.08), insulin treatment (AOR, 2.50; 95% CI, 1.91–3.28), glucocorticoid use (AOR, 1.53; 95% CI, 1.18–1.98), need for mechanical ventilation (AOR, 1.94; 95% CI, 1.43–2.64), need for renal replacement therapy (AOR, 1.99; 95% CI, 1.48–2.67), total parenteral nutrition (AOR, 1.34; 95% CI, 1.12–1.73), duration of diabetes (AOR, 1.04; 95% CI, 1.01–1.08), and procalcitonin level (AOR, 1.004; 95% CI, 1.001–1.008) were significantly associated with increasing severity of dysglycemia in critically ill patients. In contrast, patients admitted with a higher serum albumin level (AOR, 0.95; 95% CI, 0.93–0.97) were less likely to have dysglycemia during ICU stay.

**Table 3:** Multivariate analysis of factors associated with dysglycemia in critically ill patients

Dysglycemia as an ordinal categorical variable (0, 1, 2, 3)	OR	95% CI	*p* value
APACHE II, per 1 increment	1.06	1.05–1.08	< 0.001
Insulin use			
No	1 [reference]		
Yes	2.50	1.91–3.28	< 0.001
Treated with corticosteroids			
No	1 [reference]		
Yes	1.53	1.18–1.98	< 0.001
Need for MV			
No	1 [reference]		
Yes	1.94	1.43–2.64	< 0.001
Need for RRT			
No	1 [reference]		
Yes	1.99	1.48–2.67	< 0.001
SA, per 1 g/L increment	0.95	0.93–0.97	< 0.001
TPN			
No	1 [reference]		
Yes	1.34	1.12–1.73	0.01
DOD, per 1 year increment	1.04	1.01–1.08	0.01
PCT, per 1 ng/ml increment	1.004	1.001–1.008	0.02

DOD, duration of diabetes (years); MBG, mean blood glucose level; MV, mechanical ventilation; RRT, renal replacement therapy; IT, insulin treatment; SA, serum albumin; TPN, total parenteral nutrition; GU, glucocorticoids use; PCT, procalcitonin

## Discussion

In the present study, we investigated the association between 3 domains of glucose control and mortality in acutely ill patients depending on their diabetes status. The key findings are as follows. GV was independently associated with increased mortality among critically ill patients. Its adjusted effect on mortality was similar among patients of both diabetes groups. Hypoglycemia was independently and strongly associated with mortality in the non-DM group; however, its effect attenuated to non-significance in the DM group after adjusting for confounders. Although the effect of hyperglycemia (MBG > 7.8 mmol/L for non-DM patients; > 10 mmol/L for DM patients) on ICU mortality was nonsignificant, particularly after adjustment for disease severity, the association between dysglycemia (hyperglycemia, hypoglycemia, and increased GV) and mortality was cumulative in both cohorts. Compared to patients without dysglycemia, those with the 3 types of dysglycemia had nearly 3- and sixfold higher odds of mortality in the DM and non-DM groups, respectively. A higher APACHE II score, intravenous insulin infusion, glucocorticoid use, need for mechanical ventilation, need for renal replacement therapy, lower serum albumin level, total parenteral nutrition, longer duration of diabetes, and a higher procalcitonin level were significantly associated with the 3 domains of dysglycemia in critically ill patients.

These findings support the critical role of the extended concept of glucose control, which should include the 3 dimensions (hyperglycemia, hypoglycemia, and GV) and patient diabetes status into consideration.

Several observational studies have reported the association between dysglycemia (hyperglycemia, hypoglycemia, and GV) and mortality in patients admitted to the ICU [3, 4, 11]. In addition, recent studies have reported that associations between glycemic metrics and adverse outcomes varied among patients with or without diabetes [7, 9, 11, 12, 14, 21]. However, the results of these studies are inconsistent, which can partially be explained by a lack of adjustment for significant confounding factors such as comorbidities, insulin infusion, use of glucocorticoids, and hemodialysis in these studies. In our work, after adjusting for all the confounders we selected, the robust regression model showed that the effects of dysglycemia on mortality in critically ill patients differed between DM and non-DM patients.

Previous studies have suggested that GV has a more pronounced effect on mortality in patients with DM than in those without [6, 14, 25, 26]. In contrast, a recent multicenter study reported that the independent effect of GV on mortality was unaffected by diabetes status or even by HbA1c level [27]. Consistent with this finding, our results show that GV had a similar and independent effect on mortality in both DM and non-DM groups. Its effect on mortality did not reduce significantly even after adjustment for hypoglycemia and hyperglycemia. Given studies have reported that the effect of GV on mortality can be attenuated by the interaction between hypoglycemia and mortality in the non-diabetic group [27, 28], we believe that the explanation could be our selection of relatively comprehensive confounders, including multiple glucose metrics, comorbidities, disease severity, and in ICU treatment, which were added to the regression model step by step for adjustment.

In accord with most previous studies, our study reported that hypoglycemia, defined as a minimum BG < 3.9 mmol/L, has the strongest effect on mortality among the 3 dimensions of glucose control and is independently associated with high mortality in critical patients without diabetes [28, 29]. In addition, results of this study showed that this deleterious effect in patients with diabetes was significant in our initial model but attenuated and turned nonsignificant after adjustment for comorbidities, ICU treatment and disease severity. Similarly, a prospective study also showed that glucose level fluctuations in patients with diabetes are more likely to be strongly associated with adverse outcomes than hypoglycemia [30]. In addition, findings from observational studies have shown that when exposed to hypoglycemia, those with higher HbA1c levels (poor glycemic control) before admission had a lower mortality rate [14].

Hyperglycemia is a well-known marker of disease severity, the association between hyperglycemia and mortality in ICU has been reported in many studies [6, 31]. Per recent studies, the association between hyperglycemia and mortality in patients admitted to the ICU is more pronounced in patients without diabetes than in patients with diabetes [12, 32]. Our univariate analysis of hyperglycemia (MBG ≤ 7.8 mmol/L for non-DM and ≤ 10 mmol/L for DM) was consistent with these results. However, this association attenuated to non-significance in both groups after adjustment for disease severity. A plausible explanation for this observation is that the association between hyperglycemia and mortality is most affected by acute stress response, particularly when adjusting for the effect of diabetes [33]. In line with this observation, recent studies reported that hyperglycemia is not significantly associated with mortality after adjustment for disease severity [31, 34].

Moreover, our results showed that abnormalities in > 1 domain of glycemic control (including hyperglycemia) had a cumulative effect on mortality both in DM and non-DM groups, which is consistent with results of other studies [25].

Dysglycemia is defined as a deviation or fluctuations of blood glucose levels from the normal levels [11]. This common metabolic dysfunction in ICU could partly be explained by stress response, which involves several cellular pathways, such as those related to oxidant stress, immunity, and cellular homeostasis [35]. However, associations between diabetes status and nutritional condition and use of some medications (insulin/steroids) are complex [35] and can lead to poor glycemic control through increased insulin resistance and reduced β-cell secretory function.

Given the disadvantages of poorly controlled glycemic metrics, we believe identifying patients at high risk for manifested dysglycemia is crucial to a more personalized approach for targeted therapy. However, the factors associated with ICU dysglycemia are complex [36]. We simply divided them into endogenous factors (such as age, sex, disease severity, the function of pancreatic beta-cell) and exogenous factors (such as surgical trauma, nutrition therapy, insulin infusion therapy, and use of glucocorticoids) according to previous studies [34, 37].

After adjusting for covariates, severity of illness (marked as APACHE II score) had the strongest effect on dysglycemia among all the endogenous factors, followed by duration of diabetes. Prolonged diabetes can lead to worsening dysglycemia through increased insulin resistance and reduced β-cell secretory function [38]. Elevated procalcitonin level, used as a clinical marker for infection or inflammation in critically ill patients, was significantly associated with dysglycemia. Of interest, the negative correlation between serum albumin and dysglycemia suggested that nutritional status may probably help stabilize glycemic control.

Per our multivariate analysis, insulin use was the most critical factor determining the severity of dysglycemia, followed by renal replacement therapy and mechanical ventilation. Corticosteroid use also had a significant effect on dysglycemia. Total parenteral nutrition therapy during ICU appeared to play a relatively minor role in glycemic control in critically ill patients than other exogenous factors. The sample size of this study limited the evaluation of the relative effects of various dose ranges of exogenous factors on glycemic control.

Glucose level management in ICU is a key clinical concern yet the optimal glucose level target remains unclear. Our results provide further understanding of the 3 common domains of glycemic control, showing that glycemic control in critically ill patients should be in consideration of the patient’s diabetes status. Considering the increasing body of evidence highlighting the varying associations between dysglycemia domains and mortality in critically ill patients, we reckon future critical illness guidelines for glycemic control targets will recommend a personalized approach [39]. Although the causal relationship between adverse outcomes and poorly controlled dysglycemia or its 3 domains remains unclear, appropriate control of glycemic metrics has been considered to beneficial for critically ill patients [17, 35, 40, 41], and appropriate control of glycemic metrics is associated with better short-term and long-term outcomes. Further prospective cohort studies and randomized controlled trials are required to validate our findings.

A strength of our study is the comprehensive selection of confounding variables that allowed estimating the association between multiple glucose metrics, diabetes status, and mortality accurately, which was lacking in previous studies to our knowledge. We stratified patients into 2 groups and used 3 models adjusting for confounders, which allowed reliably estimating the effect of each glucose metric on mortality. Another strength lied in defining dysglycemia as an ordinal categorical variable, determining its cumulative effect on mortality, and further identifying its risk factors.

Our study had some limitations. First, this was a single-center study. Although the patient population was heterogeneous (patients admitted with a variety of medical and surgical diagnoses), it is not completely representative of all critically ill patient population.

Second, given the retrospective study design, selection bias and misclassification cannot be excluded. Because physicians in our ICUs do not routinely request HbA1c measurements without a clinical suspicion of diabetes, we only used electronic medical records to identify DM status. Therefore, we could not determine whether patients with undiagnosed diabetes were included and could not further analyze stress-induced hyperglycemia and diabetes-induced hyperglycemia, which required HbA1c measurements [32, 33, 42].

Furthermore, we could not determine the type of DM, which is critical given that significant differences may exist between DM types. Considering a recent study focusing on higher GV in critically ill patients with type 1 DM [43], we hope further research will be conducted on personalized ICU glucose control strategy for the different diabetes types. In addition, observational studies have found that different nutritional provisions and therapies are associated with an increased risk of dysglycemia and adverse outcomes [44]. However, because of a limited scope of the medical records used in our study, the nutrition and insulin infusion data extracted were dichotomous variables used only for adjustments and further identification of risk factors of dysglycemia, we could not further determine the precise insulin dose and energy intake, which have been shown to affect dysglycemia [44]. Further research with individual-level data on these confounders may help clarify these questions.

## Conclusion

This retrospective single-center observational study demonstrated that dysglycemia occurring during ICU admission was associated with mortality in all patients. However, the associations between the 3 domains of glycemic control and mortality in the ICU varied by diabetes status. Hypoglycemia had the strongest association with mortality, particularly among those without diabetes; however, GV was associated with mortality in ICU for all patients. Finally, the effect of hyperglycemia was not significant after adjustments for confounders. Of importance, the 3 glycemic metrics tended to have a cumulative association with mortality. Although the causal relationship between dysglycemia and mortality remained unclear, our findings have implications for current glucose control.  Glucose management in critically ill patients should be specific to the patient’s need considering the diabetes status and broader dimensions.

## Data Availability

The datasets used and analysed during the current study are available from the corresponding author on reasonable request.
