# Correction to: Association between dysglycemia and mortality by diabetes status and risk factors of dysglycemia in critically ill patients: a retrospective study

**DOI:** 10.1007/s00592-022-01853-8

**Published:** 2022-03-01

**Authors:** Haoming Ma, Guo Yu, Ziwen Wang, Peiru Zhou, Weitao Lv

**Affiliations:** 1 School of Nursing, Jinan University, No. 601, West Huangpu Avenue, Tianhe District, Guangzhou City, Guangdong Province China; 2 Health Management Centre, The First Affiliated Hospital of Jinan University, No. 613, West Huangpu Avenue, Tianhe District, Guangzhou City, Guangdong Province China; 3 Division of Critical Care, The First Affiliated Hospital of Jinan University, No. 613, West Huangpu Avenue, Tianhe District, Guangzhou City, Guangdong Province China

## Correction to: Acta Diabetologica 10.1007/s00592-021-01818-3

Authors would like to update below as article note which was missed out in original publication. The original article has been corrected.

Haoming Ma and Guo Yu contributed equally to this manuscript.

